# Injury incidence and burden during senior inter-provincial field hockey tournaments

**DOI:** 10.17159/2078-516X/2021/v33i1a11832

**Published:** 2021-12-01

**Authors:** N Pereira, TL Burgess, L Corten

**Affiliations:** 1 Division of Physiotherapy, Faculty of Health Sciences, University of Cape Town, South Africa; 2 Centre for Medical Ethics and Law, Faculty of Medicine and Health Sciences, Stellenbosch University, Cape Town, South Africa; 3 Division of Physiotherapy, School of Health Sciences, University of Brighton, United Kingdom

**Keywords:** injury burden, injury prevention, gender, South African field hockey

## Abstract

**Background:**

Field hockey is an Olympic sport played internationally and in which South Africa is a participating nation. It also has its own world cup. Few injury studies have been published on South African field hockey. Research efforts should increase within the sport to ensure safe participation and mitigate the inherent injury risks.

**Objectives:**

The objective of the study was to attend the male and female inter-provincial field hockey tournaments in South Africa and determine the incidence of injury and burden of acquired sport injuries (time-loss and medical attention).

**Methods:**

A quantitative, descriptive, longitudinal study, including 133 females and 139 males, was conducted. Participants completed baseline questionnaires prior to the tournament and post-match questionnaires detailing injuries during the tournament.

**Results:**

The recorded injuries were 77.9 (females) and 99.5 (males) per 1 000 player match hours. Medical attention was 51.9 (females) and 70.3 (males) injuries per 1 000 player match hours. The result for time-loss injuries was 4.3 (females) and 7.5 (males) injuries per 1 000 player match hours.

**Discussion:**

The study found high incidence rates of all injuries and medical attention injuries; however, the incidence of time-loss injuries was low in comparison to existing literature. Comparing current results to existing literature is challenging due to the heterogeneity of methodologies and injury definitions in field hockey research.

**Conclusion:**

This was the largest observational study in field hockey conducted in South Africa. The international sporting body should establish a consensus for future research and the South African Hockey Association explore long-term surveillance in South Africa to mimic similar national codes.

Field hockey is an Olympic discipline played widely across the world at various levels.^[1]^ In South Africa, the sport is growing with increasing participation from primary and secondary school to senior provincial level. It is a high-speed team sport, consisting of short bursts of sprinting with technical coordination of a stick and ball, while executing a coach’s tactical game plan. ^[2–4]^ The senior inter-provincial tournament (IPT) is the elite level of South African field hockey and takes place annually. Teams play several matches over a period of a week, including a round robin, playoffs, and a final. The tournaments observed in this study were held separately during 2018 in KwaZulu Natal. From this tournament, the national teams are selected for international events, including the World Cup and Olympic Games.

To date, there are few published studies which have been undertaken in South Africa exploring injury incidence. The existing research is challenging to compare due to the heterogeneity of research methodologies. Research into a local female provincial hockey season during 2004 reported injury incidence to be 6.3 per 1 000 hours played.^[5]^ Injury data collected from the International Federation for Field Hockey (FIH) tournaments report the incidence of injury to be 26.0–29.1 per 1 000 hours for females and between 20.8–90.9 per 1 000 hours for males.^[1]^ The definitions used in these studies are in stark contrast, making the comparison regarding injury incidence challenging.

Research in field hockey in South Africa is limited when compared to football and rugby due to a lack of funding and the amateur status of the sport. ^[6–7]^ There is currently no data available on injury incidence to male South African field hockey players, and no incidence literature for field hockey has been published in South Africa since 2007. There are no associated statistics established for this population; therefore, the aim of this study was to observe the current injury incidence and burden in South African field hockey, as well as the association of previous injury and gender with incidence by conducting this study at the annual IPT.

## Methods

### Ethical approval

The study was approved by the University of Cape Town’s Faculty of Health Sciences Human Research Ethics Committee (HREC REF: 117/2018) and by the CEO of the South African Hockey Association (SAHA).

### Study design and setting

The study was quantitative, descriptive, and longitudinal. It took place at two separate tournaments in 2018, namely, the male and female IPTs held in Pietermaritzburg and Durban, respectively. All players attending these tournaments were invited to enrol in the study.

### Procedure

Identification of participants was carried out by contacting SAHA and subsequently, the provincial unions. Informed consent forms and baseline questionnaires were disseminated by the participating unions. Participants who did not respond to the research invitation were approached and recruited during a pre-tournament briefing at the venue in the days preceding commencement. Participants were excluded if they were under the age of 18 years or did not provide consent to be involved in the study.

### Measurement instrument

Players were asked to complete a self-designed questionnaire prior to the tournament to gather background information such as age and previous injury history. During the tournament, the players were also asked to complete daily self-reporting of injuries after each match using a digital injury reporting form. The results from both questionnaires were collated, de-identified, and coded for analysis.

As there is currently no consensus on injury surveillance for field hockey. The questionnaires were based on the BokSmart injury surveillance forms, a questionnaire used in the South African Rugby Union injury surveillance report.^[7]^ The injury definition was adapted from the Federation Internationale de Football Association (FIFA) medical assessment and research centre. ^[7, 8]^ The following definition was used in this study: ’Any physical discomfort resulting from hockey-related activity, regardless of severity or need for medical attention as perceived by the player. ‘^[8]^ Injuries were categorised into one of three categories: “all injuries”, includes any and all physical complaints reported by participants regardless of severity or need for medical attention; “medical attention injuries”, injuries requiring evaluation or treatment from a health professional; and “time-loss injuries”, injuries that cause inability to participate in training or matches.

### Statistical analysis

Incidence was calculated as follows: each team had 11 players on the field during a match, with a total match duration of one hour (four 15-minute quarters). The tournament was played over seven days and regardless of progression through the tournament, each team played on all seven match days. Player-match-hours was therefore calculated as: 11 players × 7 days × 1h per game = 77 player-match-hours. Incidence is presented as injuries per 1 000 player- match hours, with 95% confidence intervals.

The descriptive data were not normally distributed based on the Shapiro-Wilk test; therefore, the data are presented as median and interquartile ranges. Associative analysis in statistics was performed using the chi-squared test, odds ratio, and risk ratio to assess previous injuries and incidence, and to compare the male and female tournament incidence. Statistical analyses were performed using Statistical software IBM SPSS version 25 (Armonk, NY: IBM Corp).

## Results

### Study sample

The study enrolled 272 participants (133 females, 139 males) attending the 2018 IPT. The players’ ages across the two tournaments were not normally distributed (W = 0.90, p < 0.01). The ages of participants showed a median (interquartile range) age of 22 (20–26) years for males and 21 (19–25) years for females.

**Fig. 1:**
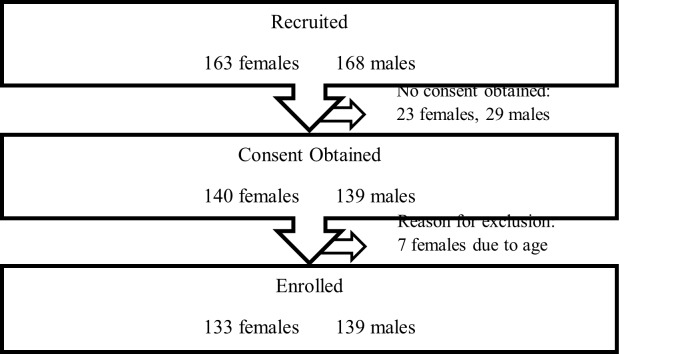
Recruitment flowchart

In the female tournament, 163 participants completed the tournament injury reporting form; 23 participants did not provide informed consent and their data were excluded. Seven participants were excluded due to being underage. In the male tournament, 168 participants completed the injury reporting form, but 29 did not complete the consent form and their data were excluded from the study. The recruitment flowchart is presented in Fig. 1.

### Response rate

**Fig. 2:**
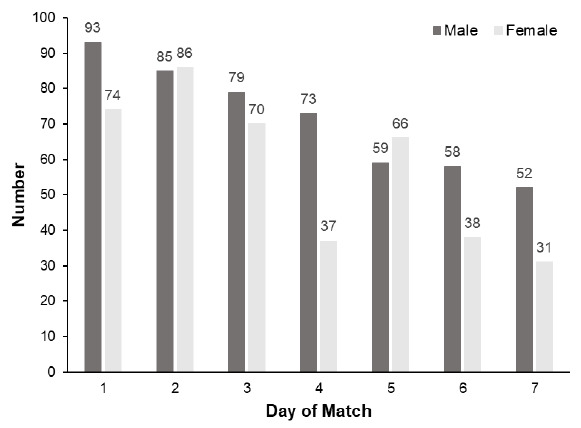
Number of injury report forms per match day

The results relied on the participants completing daily injury report forms. The variation in response rate per day is presented in Fig. 2. The male participants’ reporting began with 93 responses on day one (67% of recruited sample), and steadily decreased to 52 (37% of recruited sample) on the final day of the tournament. The female participants’ reporting began with 74 responses (55% of recruited sample), increased to 86 (64% of recruited sample) on day two, and then decreased to 70 (52% of recruited sample) for day three.

### Incidence of injury

In the female tournament, a total of 72 injuries were reported, with 48 requiring medical attention, and four resulting in time-loss (Table 1). The incidence calculated per 1 000 player match hours was 77.9 (95% CI: 47.1 – 108.1) for the overall female tournament, with 51.9 (95% CI: 32.3 – 71.5) injuries per 1 000 player match hours requiring medical attention, and 4.32 (95% CI: 1.04 – 9.7) injuries per 1 000 player match hours resulting in time-loss. It should be noted that teams are anonymised and numbered one to thirteen. The same numbers from both male and female tournament represent the same province. Numbers twelve and thirteen represent different provinces.

A total of 92 injuries were reported in the male tournament, with 65 injuries requiring medical attention and seven resulting in time-loss. The incidence of injuries for the male tournament was 99.5 (95% CI: 71.9 – 127.1) injuries per 1 000 player match hours. The incidence of medical attention needed was 70.3 (95% CI: 46.1 – 94.4) injuries per 1 000 player match hours, and the incidence of time-loss was 7.5 (95% CI: −0.7 – 15.75) injuries per 1 000 player match hours (Table 2).

### Association of injuries

There were several associative statistics explored in the original study; however, previous injury as well as gender differences for injury have been outlined in Table 3. There is a statistically significant association for players who reported injury in the pre-tournament and during the tournament. There are no statistically significant associations between injuries in the male and female tournaments respectively.

## Discussion

### Incidence of injury

The primary aim of this study was to investigate the incidence of injury in field hockey during the senior male and female provincial tournaments of 2018. The injury incidence rates found in this study (99.5 and 77.9 injuries per 1 000 player match hours for males and females respectively) are higher than those found in literature investigating the FIH international tournaments of 2013 ^[1]^. It was found that injury incidence in this study ranged between 26.0–29.1 per 1 000 player match hours for females and between 20.8–90.9 per 1 000 player match hours for males. There are significant methodological and contextual differences between the present study and the FIH tournaments literature, namely in the injury definitions used and the classification of injuries.

The present study captured all self-reported injuries regardless of subsequent time-loss or the need for medical attention and is thus expected to observe higher incidence rates. The 2013 FIH tournament’s study captured injuries that required stoppage in play, resulting in the removal of the player from the field.^[1]^ Furthermore, the FIH study captured injuries recorded by match officials, with players and medical staff not involved in the study. The number of injuries resulting in time-loss or players receiving medical attention was not reported in the FIH study.

The only other published South African study on the incidence of injury in field hockey investigated ankle strength, proprioception, and injury incidence in a sample of 47 female provincial hockey players.^[5]^ Although their method of capturing injuries also utilised self-reporting, their injury definition only included injuries resulting in time-loss from field hockey for five or more days and relied on participant recall post season. Their incidence of injury of 6.32 per 1 000 player match hours is partially comparable with the present study’s time-loss injury incidence of 4.3 per 1 000 player match hours for females. However, it is inappropriate to draw meaningful comparisons between the two studies due to differences in study methodology. The incidence of time-loss injuries in the male tournament cannot be compared to other research in South Africa, as no available studies have investigated injury incidence in males.

### Injury burden

The present study observed a decrease from self-classified injuries to medical attention injuries, and then to time-loss injuries. The burden of time-loss injury was low in both tournaments with females reporting 4.32 per 1 000 player match hours and 7.5 per 1 000 player hours for males. This supports the views of international research that time-loss from field hockey is relatively low.^[9]^ A study observing American female collegiate field hockey players over a ten-year period reported an overall injury incidence of 5.36 per 1 000 player hours (training and competition), and 8.49 per 1 000 player match hours for competition only.^[10]^ Injuries in this study were defined as injury caused by field hockey, requiring medical attention and resulting in at least one day of missed training or match participation. Although the definition of injury in this study varies, there is a comparable time-loss incidence rate with the female tournament of the present study. A hypothesis for the injury/time-loss mismatch is the competitive nature of athletes, and the level of participation in the present study.^[13]^ After participating in the senior IPT, the male and female national teams are selected. This may have led to participants ’playing through injury.’ ^[3]^ There is no association between ’playing through injury’ and match outcomes, although there are certainly long-term health concerns for participants who use this approach.

Medical attention and time-loss injuries observed were lower during the female tournament compared to that of the males; however, no statistically significant differences were found regarding the injury burden between the tournaments. The overall number of self-reported injuries was considerably high, which suggests that future research should also investigate injuries not resulting in time-loss. This may suggest that the large amount of self-reported injuries was insignificant as they do not result in time-loss; however, it does indicate that players experience musculoskeletal complaints during participation, which may affect performance and is a threat to player safety.^[11]^ Sub-clinical trauma or repetitive microtrauma could be one explanation for this and should be explored in future research. Few studies in field hockey have explored the prevalence of overuse injuries. Overuse is reported at 28.7% of all injuries in female high school field hockey, and at 17% of all injuries in female collegiate field hockey.^[10]^ In male and female club hockey, it is reported that chronic/overuse injuries are 20% more frequently reported than acute injuries. ^[10, 12]^ In a German study comparing indoor and outdoor field hockey, outdoor hockey reported overuse for males and females as a combined 53.4% of all reported injuries.^[4]^ This supports the theory that the burden of overuse injuries is often greater than observed and which may account for the incidence/time-loss mismatch seen in the present study.

### Generalisability of the study results

The present study may be used as an updated record of observational injury incidence for South African field hockey, which should be improved upon over time. The injury definition used in this study, ’any physical discomfort resulting from hockey related activity, regardless of severity or need for medical attention,’ was broad enough to capture all physical complaints and to subsequently categorise them by time-loss and whether medical attention was required. This is in line with the consensus statement for football injuries, which the researchers deemed to be a sport similar to hockey in physical and tactical game play.^[8]^

The format of the tournaments in the present study included seven consecutive days of fixtures for all participating teams, regardless of playoffs and finals. This is not common practice in international field hockey, with tournaments such as the summer Olympics and World Cup taking place over several weeks. It is thus hypothesised that the format of the tournament had an influence on observed injuries for both males and females in field hockey. This has been explored in other team sports, including cricket, rugby, and football. In football, it has been reported that congested periods of fixtures can affect the match recovery of players.^[14]^ This may present challenges when comparing injury rates with other tournaments or seasonal field hockey research.

Despite the heterogeneity of definitions, methodologies, and formats in research, the researchers maintain that the results of this study are an estimate of all injuries observed in the tournament format of field hockey for senior participants within South Africa.

### Strengths and limitations

The present study included the largest sample observed in South African field hockey to date and combined both male and female participants. It is the first study in South Africa to capture male injury incidence in field hockey.

The injury incidence observed in the present study should form part of long-term injury prevention for South African field hockey participants. This is in line with the sequence of injury prevention proposed in 1992 ^[15]^, which has since been updated to include the ‘Translating Research into Prevention Practice’ framework, and the ‘Team Sport Injury Prevention’ cycle ^[16]^. Through each evolution of the injury prevention paradigm, injury surveillance remains the foundation on which prevention or risk reduction is built.

The definition of injury utilised in the present study is both a strength and a limitation. It allowed the researchers to capture all injuries experienced by participants because of its all-encompassing definition, which is a strength. Much of the current literature captures only injuries causing time-loss or removal from field of play as their identification of injury.^[1]^

The use of time-loss in the identification of injury would have yielded a limited amount of data compared to what was collected with this study’s definition. The use of self-reporting allowed the study to explore a large sample, albeit with limited resources. This method, combined with the broad injury definition, allowed the study to observe injury and its association through a wide lens. In the current context of this research being the first of its kind in South Africa, an overview was warranted so that subsequent research can further explore the main findings of this study.

The use of a self-reporting questionnaire as the method of injury reporting is a limitation to the present study.^[17]^ The subjectivity of self-reported injuries can lead to over- or under-reporting of injuries, and although participants were guided by the injury definition, there remained a constant potential input error.

The completion rate of the injury questionnaire was also a limitation to this study, and during both tournaments, responses declined throughout the week. This limited the ability of the data collected to reflect the actual injury incidence of the players during the tournaments, as it assumed that the non-responses represented non-injury. This poses a challenge for future research to create a follow-up protocol or to use third-party data collection to mitigate the decline in the response rate as a tournament progresses.

The short observation period (seven-day tournament), combined with the study’s inclusive injury definition and self-reporting design, may have led to overreporting of the incidence of injury compared to that in existing literature. Furthermore, this may have led to the underestimation of the role of overuse injuries being part of the high incidence rate recorded. Although it was postulated that certain injuries not resulting in time-loss would fit into an acute-on-chronic pattern or be caused by previous injury or overuse, the researcher cannot objectively comment on this based on the limitations in the classification of injuries. The study was also unable to categorise injury mechanisms, and it is unknown whether contact or non-contact mechanisms caused the injuries observed. The use of injury classification systems, such as the Oslo Sports Trauma Research Questionnaire ^[18]^ or the Orchard Classification System, ^[19]^ could have strengthened the external validity of the study.

### Recommendations for future research

The researchers recommend the exploration of web-based surveillance for future research.^[3]^ This method is already employed by national bodies for cricket and rugby within South Africa ^[17]^ and would allow SAHA to observe patterns of injury over extended periods of time in different formats of the game. Internationally, a clear consensus statement for field hockey research should be issued by the FIH to standardise future research. ^[4, 9, 10, 12, 20]^

## Conclusion

To our knowledge, this was the largest observational study conducted in South African field hockey that included both male and female players. High incidence rates of all injuries and medical attention injuries were identified; however, the incidence of time-loss injuries was low in comparison to existing literature.

## Figures and Tables

**Table 1:** Female tournament injury incidence

Team	All injuries	Incidence per 1 000 player match hours	Medical attention needed	Medical attention incidence per 1 000 player match hours	Time- loss	Incidence of time-loss per 1 000 player match hours
**1**	10	129.8	7	90.9	0	0
**2**	4	51.9	3	38.9	0	0
**3**	10	129.8	5	64.9	1	12.9
**4**	0	0	0	0	0	0
**5**	5	64.9	2	25.9	0	0
**6**	8	103.8	8	103.8	2	25.9
**7**	6	77.9	6	77.9	0	0
**8**	5	64.9	4	51.9	0	0
**9**	2	25.9	1	12.9	0	0
**10**	5	64.9	3	38.9	0	0
**11**	4	51.9	4	51.9	0	0
**12**	13	168.8	5	64.9	1	12.9

**Total**	72	77.9 (95% CI: 47.1–108.1)	48	51.9 (95% CI: 32.3–71.5)	4	4.3 (95% CI: −1.04–9.7)

Injury data are presented as individual team and total injury numbers and incidence rates per 1000 player match hours. Injuries were categorised into one of three categories: “all injuries”, includes any and all physical complaints reported by participants regardless of severity or need for medical attention; “medical attention injuries”, injuries requiring evaluation or treatment from a health professional; and “time-loss injuries”, injuries that cause inability to participate in training or matches. Each team number represents a provincial team, team 13 did not participate in the female tournament.

**Table 2:** Male tournament injury incidence

Team	All injuries	Incidence per 1 000 player match hours	Medical attention needed	Medical attention incidence per 1 000 player match hours	Time- loss	Incidence of time-loss per 1 000 player match hours
**1**	5	64.9	2	25.9	0	0
**2**	4	51.9	3	38.9	1	12.9
**3**	8	103.8	4	51.9	0	0
**4**	10	129.8	5	64.9	0	0
**5**	12	155.8	7	90.9	2	25.9
**6**	2	25.9	2	25.9	0	0
**7**	10	129.8	8	103.8	0	0
**8**	13	168.8	11	142	1	12.9
**9**	8	103.8	7	90.9	0	0
**10**	5	64.9	4	51.9	0	0
**11**	9	116.8	9	116.8	3	38.9
**13**	6	77.9	3	38.9	0	0

**Total**	92	99.5 (95% CI: 71.9 – 127.1)	65	70.3 (95% CI: 46.1 – 94.4)	7	7.5 (95% CI: −0.7 – 15.8)

Injury data are presented as individual team and total injury numbers and incidence rates per 1000 player match hours. Injuries were categorised into one of three categories: “all injuries”, includes any and all physical complaints reported by participants regardless of severity or need for medical attention; “medical attention injuries”, injuries requiring evaluation or treatment from a health professional; and “time-loss injuries”, injuries that cause inability to participate in training or matches. Each team number represents a provincial team, team 12 did not participate in the male tournament.

**Table 3:** Associations with tournament injuries

Factor	Sub-category	Not injured during tournament	Injured during tournament	Statistical and p-value
**Previously injured in past year**	No	92	47	X2 = 20.645 p < 0.0001 Odds ratio (95% CI) = 3.548 (2.034 – 6.190) Risk ratio (95% CI) = 1.861 (1.375 – 2.519)
	Yes	32	58	

**Gender**	Female	56	53	X2 = 073 p = 0.390
	Male	69	52	
